# International Consensus on a Dental Antibiotic Stewardship Core Outcome Set

**DOI:** 10.1016/j.identj.2023.03.006

**Published:** 2023-04-12

**Authors:** Wendy Thompson, Leanne Teoh, Celine Pulcini, Susie Sanderson, David Williams, Vanessa Carter, Carole Pitkeathley, Tanya Walsh

**Affiliations:** a University of Manchester, Manchester, United Kingdom; b University of Melbourne, Melbourne, Australia; c Université de Lorraine, Nancy, France; d FDI World Dental Federation, Geneva, Switzerland; e Queen Mary University of London, London, United Kingdom; f Johannesburg, South Africa; g Blyth, United Kingdom

**Keywords:** Dental, Antibiotic, Antimicrobial, Prescribing, Stewardship, Outcomes

## Abstract

**Introduction:**

Antibiotic resistance is a global health crisis. Ensuring responsible, appropriate use (stewardship) is an important for keeping antibiotics working as long as possible. Around 10% of antibiotics across health care are prescribed by oral health care professionals, with high rates of unnecessary use. To maximise the value from research to optimise antibiotic use in dentistry, this study developed international consensus on a core outcome set for dental antibiotic stewardship.

**Methods:**

Candidate outcomes were sourced from a literature review. International participants were recruited via professional bodies, patient organisations, and social media, with at least 30 dentists, academics, and patient contributors in total. Outcomes scored “critical for inclusion” by >70% of the participants (dentists, academics, and patients) after 2 Delphi rounds were included in the core outcome set following a final consensus meeting. The study protocol was registered with the COMET Initiative and published in BMC Trials.

**Results:**

A total of 33 participants from 15 countries, including 8 low- and middle-income countries, completed both rounds of the Delphi study. Antibiotic use outcomes (eg, appropriateness of prescribing), adverse or poor outcomes (eg, complications from disease progression), and a patient-reported outcome were included in the final, agreed core set. Outcomes relating to quality, time, and cost were not included.

**Conclusions:**

This core outcome set for dental antibiotic stewardship represents the minimum which future studies of antibiotic stewardship in dentistry should report. By supporting researchers to design and report their studies in a way meaningful to multiple stakeholders and enabling international comparisons, the oral health profession's contribution to global efforts to tackle antibiotic resistance can be further improved.

## Introduction

Antimicrobial resistance (AMR) is a global health crisis.^1^ In 2019, more people died from infections that were resistant to antibiotics than from HIV and malaria combined.^2^ The burden is increasing rapidly and, without appropriate intervention, by 2050 10 million deaths each year are expected from resistant infections: more than from cancer.^3^ Antibiotic stewardship (ABS) can be defined as “a coherent set of actions which promote appropriate use of antibiotics, i.e. in ways that ensure sustainable access to effective therapy for all who need them.”^4^

Antibiotics are prescribed by dentists far more often than other antimicrobial drugs.^5^ An estimated 10% of antibiotics prescribed across health care worldwide originate from oral health professionals, with most in primary care and community settings.^4^ High rates of unnecessary prescribing have been demonstrated in dentistry, including more than 80% inappropriate for prophylaxis in the US^6^ and more than 80% not in accordance with guidelines in the UK.^7^ The essential role of the oral health profession in efforts to tackle antibiotic resistance is increasingly being recognised internationally.^4^

A core outcome set (COS) is a minimum set of outcomes that should be reported by all studies on a particular topic.^8^ Agreement on a standardised COS allows collation of results across research studies by increasing homogeneity between studies and care settings and reducing bias in outcome reporting. In addition to the core outcomes, individual studies must supplement the COS with additional measures to meet their specific study aim.

Relatively few trials of ABS interventions have, to date, been conducted in dental compared with medical settings.^9^^,^^10^ A dental ABS COS is important, therefore, to facilitate meaningful comparisons between studies and to enable the oral and dental profession to make an effective contribution to global efforts tackling antibiotic resistance. Establishing the COS before an anticipated growth in the number of studies about dental ABS occurs will ensure derivable benefit from them can be maximised.

This study aimed to develop international consensus on a core outcome set for dental antibiotic stewardship (COS-DABS) covering both therapeutic and prophylactic use. COS-DABS is intended for use in studies evaluating ABS interventions in primary care, community, and outpatient dental settings.

## Methods

In accordance with guidance from COMET,^11^ this paper follows the Core Outcome Set-STAndards for Reporting: the COS-STAR Statement.^12^

### Protocol/registry entry

Registered in the COMET database, the protocol was also published in a specialist trials journal.^13^

### Participants

People aged 18 years and older and who gave informed consent were included if they had experience of dental antibiotics as either (1) clinicians who had prescribed them, (2) academics interested in dental antibiotic stewardship, or (3) people with lived experience of them for a dental (therapeutic or prophylactic) reason.^13^ Anyone younger than 18 years old, those unable to consent, and those with no lived experience of antibiotics were ineligible to participate.

The plan was to recruit at least 30 participants to the study: 10 clinicians, 10 academics, and 10 people with lived experience of antibiotics as either a dental patient or their parent/carer. This was a pragmatic choice to maintain equity amongst the groups whilst taking into account the relatively few clinical and academic experts working internationally on dental antibiotic stewardship.^11^ At least 10 to 18 per group is usually recommended in the literature.^14^^,^^15^ No additional personal data to describe the characteristics of participants were collected to maintain anonymisation and depersonalisation of the results.

Convenience sampling was employed. Clinicians were recruited through professional bodies (eg, national dental associations) or social media (Twitter). Academics were recruited through the Global Antibiotics Research in Dentistry early career researcher network,^16^ Twitter, and authors of relevant publications.^17^ Adult patients and parent/carer participants were recruited through patient-representative bodies, Twitter, and word of mouth.

### Study steering group

The study steering group oversaw all aspects of the research and comprised dentists, academics, and lay members, as detailed in the protocol.^13^

### Information sources

Candidate outcomes for the consensus exercise were identified based on previous studies, with additional items identified by study steering group members.^13^ Outcomes identified by the steering group as not relevant to primary dental care or outpatient settings were excluded. Although the aim of antibiotic stewardship is to reduce the development and spread of antibiotic resistance, suitable measures relating to colonisation or infection with resistant bacteria for routine use in ABS studies have not yet been published.^18^ For this reason, microbiologic outcomes were not included in the long list.

### Consensus process

As described in the study protocol, consensus was sought using a 9-point Likert scale.^13^ Participants rated the importance of each candidate outcome using the DelphiManager system. Two rounds of an online Delphi survey were undertaken, with participants invited to suggest additional outcomes. Any potential additions suggested by 2 or more participants in round 1 were included for rating by all participants in round 2 of the Delphi.

Those who completed both Delphi rounds were invited to a final consensus meeting to review the results from the Delphi study and finalise the core outcome set. The meeting was held online and included deciding how to address missing data for outcomes and conflicts in the results between participant groups. Study steering group members were also invited to the final consensus meeting. As set out in the study protocol, the meeting needed to include at least 2 clinicians, 2 academics, and 2 people with lived experienced of dental antibiotics to ensure equal representation amongst the groups.^13^

### Outcome scoring

During the Delphi study, each participant rated each candidate outcome by selecting a number between 1 and 9 to indicate its importance. A score of 1 to 3 indicated “limited importance,” 4 to 6 indicated “important,” and 7 to 9 signified “critically important” outcomes. Participants also had the option to select “unable to rate.” Scores were summarised for each participant group to show how many scored each potential outcome as “critically important” or “unimportant.”

### Consensus definition

Outcomes scored as “critically important” by >70% of participants in the Delphi study were considered suitable for inclusion in the final COS, in accordance with standard COS methods.^19^ Outcomes rated as “critical” by <50% or “unimportant” by >70% were excluded from the COS. Other combinations indicated “no consensus.” The rationale is that the majority should feel the outcome critical for inclusion and only a minority should consider it to have little or no importance.^11^

All outcomes identified as suitable for inclusion in the final COS were reviewed at the final consensus meeting. If more than 5 of the outcomes were rated as “critical” for inclusion in the core set, the steering group had the option to apply a higher threshold of 75% and 25%, respectively. During the final consensus meeting, each outcome was voted on following discussion amongst the participants. Outcomes were included in COS-DABS if voted for by >70% of those present, with at least one from each participant group (clinician, academic, and patient/parent/carer).

### Ethics/consent

Ethical approval was from the University of Manchester Research Ethics Committee proportional review process (ref. UREC 2021-11905-20268 dated 2 August 2021 and amendment approved 6 September 2021).

All participants provided informed consent before participating in the Delphi study, by checking the box in the online DelphiManager system: “I agree to participate in and receive email notifications regarding this study.” The invitation for the final consensus meeting reminded participants that attendance indicated consent to participate. This was reiterated verbally at the meeting.

## Results

### Protocol deviations

The number of participants completing the Delphi Study (n = 33) deviated slightly from protocol (n = 30) due to differences in the recruitment and retention rates between the groups (see Table 1). The steering group assessed this as having minimal impact on the results.

**Table 1:** Recruitment and retention through the COS-DABS Delphi study.

	Clinicians	Academics	Patients
Consent to participate	13	15	11
Completed round 1	12	14	10
Completed round 2	11	14	8

The final consensus meeting's mix of participants also deviated slightly from the protocol, which specified a minimum of 2 from each participant group to attend. In the event, 2 clinicians, 3 academics, and 1 person with lived experience as a patient, who had completed the Delphi study, attended the meeting. In addition, 3 members of the study's steering group (1 clinician, 1 academic, and 1 patient) also attended. The study steering group members agreed that involvement of the steering group patient-representative alongside the patient participant who had completed the Delphi study would ensure a strong patient voice and equity between participant groups, thus minimising its impact on the results.

### Participants

A total of 39 participants experienced with dental antibiotics (13 clinicians, 15 academics, and 11 patients/parents/carers) were recruited to the study in September 2021. Round 1 of the Delphi study took place in October 2021 and round 2 was in November 2021. The numbers of participants completing each stage of the Delphi study are detailed in Table 1. Participants from 15 countries took part, including from 7 high-income countries (Austria, Canada, Ireland, Italy, Switzerland, UK, and US) and 8 low- and middle-income countries (Costa Rica, Ghana, Nigeria, Serbia, South Africa, Tanzania, Tunisia, and Turkey).

The final consensus meeting, in December 2021, was attended by 6 participants who had completed the Delphi study together with 3 members of the study steering group.

### Outcomes

The candidate outcomes included in the Delphi study are included in Table 2 and detailed further in the study protocol.^13^ No additional outcomes were included, as none of the 8 additional outcomes suggested by participants during round 1 was proposed by a second participant.

**Table 2:** Delphi data and final consensus: summary of the Delphi round 2 ratings and final consensus meeting decision for each candidate outcome, showing the percentage of all participants (and of each stakeholder group) scoring each outcome as critical.^7^, ^8^, ^9^

	Overall (%)		Stakeholder group (%)	Final consensus
**Antibiotic use**				
Appropriateness of antibiotic prescribing	100			IN
		Clinicians	100	
		Academics	100	
		Patients	100	
Rate of antibiotic prescribing	91			IN
		Clinicians	100	
		Academics	79	
		Patients	100	
No. of antibiotics prescribed	85			IN
		Clinicians	92	
		Academics	79	
		Patients	86	
**Adverse or poor outcomes**				
Serious adverse outcomes	94			IN
		Clinicians	92	
		Academics	100	
		Patients	86	
Complications or harm resulting from disease progression	91			IN
		Clinicians	92	
		Academics	86	
		Patients	100	
Complications or harm resulting from antibiotic treatment	85			IN
		Clinicians	83	
		Academics	86	
		Patients	86	
Need for escalation of care	82			IN
		Clinicians	83	
		Academics	79	
		Patients	86	
Complications or harm resulting from surgical site (wound) infection	73			NO CONSENSUS
		Clinicians	75	
		Academics	57	
		Patients	100	
Complications or harm resulting from distance site infection (elsewhere in the body)	52			NO CONSENSUS
		Clinicians	58	
		Academics	36	
		Patients	71	

**Patient-reported measures**				

Ability to carry on with daily life as normal	73			IN
		Clinicians	83	
		Academics	57	
		Patients	86	
Satisfaction with the result (outcome) of the care provided	52			NO CONSENSUS
		Clinicians	67	
		Academics	36	
		Patients	57	
Satisfaction with the dental treatment provided	48			NO CONSENSUS
		Clinicians	67	
		Academics	29	
		Patients	57	
Time until symptom resolution (after treatment)	48			NO CONSENSUS
		Clinicians	50	
		Academics	43	
		Patients	57	
Mental health impact	36			OUT
		Clinicians	42	
		Academics	21	
		Patients	57	
Need to taking time off usual responsibilities	27			OUT
		Clinicians	25	
		Academics	21	
		Patients	43	
**Time to clinical response**				

Severity of symptoms whilst waiting for resolution (after treatment)	58			NO CONSENSUS
		Clinicians	58	
		Academics	50	
		Patients	71	
Time taken until treatment	58			NO CONSENSUS
		Clinicians	67	
		Academics	57	
		Patients	43	
No. of unplanned return dental visits (after treatment)	42			NO CONSENSUS
		Clinicians	50	
		Academics	50	
		Patients	14	

**Cost of the intervention**				

Cost to patients	58			NO CONSENSUS
		Clinicians	50	
		Academics	69	
		Patients	57	
Cost to the health care system	48			NO CONSENSUS
		Clinicians	58	
		Academics	38	
		Patients	57	
Cost to dental prescribers	33			NO CONSENSUS
		Clinicians	25	
		Academics	38	
		Patients	43	

A summary of the rating of each outcome during round 2 of the Delphi study is shown in Table 2. Where participants changed their rating of outcomes, some also shared their reasons for changing. Whilst most related to reflecting further on the issue in the light of how peers had scored the outcome, there were some interesting reflections in particular relating to the patient-reported outcomes:

• *After having infection, I could not function with daily activities.*
• *I realised it is critical that we do no harm to the body just to treat a tooth.*
• *I don't think of severe outcomes in dentistry that impact normal life—perhaps it is more important?*

### Core Outcome Set

The final core outcome set consisted of 3 main outcomes: antibiotic use, adverse or poor outcomes, and patient-reported outcomes. Sub-outcomes together with the reflections of the final consensus meeting are presented below.

#### Antibiotic Use

Sub-outcomes were appropriateness, quantity, and rate of antibiotic prescribing. Whilst “appropriateness” was the only outcome which all participants felt was critical for inclusion in the core outcome set, the participants noted it to be one of the hardest to define due to differences in clinical guidelines and the nature of dental service provision around the world. As a consequence, operationalising this outcome for designing and reporting studies of dental ABS across different contexts would be one of the most challenging.

#### Adverse or poor outcomes

Sub-outcomes were serious adverse outcomes, harm resulting from disease progression, harm resulting from antibiotic treatment, and the need for escalation of care. As clinical trials are routinely required to collect data about “serious adverse outcomes,” the consensus meeting participants recommended that trials using COS-DABS should include “distant site infections (such as infective endocarditis or *Clostridioides difficile* infection)” within the definition of “serious adverse outcomes” even though no consensus was achieved on this outcome through the Delphi study outcome.

#### Patient-reported measure

Ability to carry on with daily life as normal was the only patient-report measure identified. Many at the final consensus meeting were surprised and felt that this may be due to differences between patients receiving prophylactic antibiotics for routine care (such as to prevent infective endocarditis), compared to therapeutic antibiotics for the treatment of an active infection.

## Discussion

This COS for use in dental studies of ABS is the first across health care to have involved clinicians, academics, and patient participants. Outcomes related to antibiotic use, adverse or poor outcomes, and the patient's ability to carry on with normal daily life were identified as critical for inclusion. Further studies will be required to operationalise them, such as identifying detailed definitions for each indicator or outcome measurement instruments. Whilst COS-DABS represents the minimum set of outcomes which should be measured by all studies of dental antibiotic stewardship, researchers will need to add further outcomes specific to meet the aims of their studies.^18^ The candidate outcomes that were excluded from the COS represent a resource for researchers selecting additional outcomes for their studies. In addition, as microbiological outcomes are developed for use in ABS studies across wider health care settings, future researchers may identify suitable additional outcomes for use in dental studies.^20^

To enable comparisons of the quantity of antibiotic use across outpatient settings (including but not limited to dentistry), international consensus on a set of metrics has been published, including prescriptions per clinician contact.^21^ Previous studies of dental ABS interventions have reported only antibiotic use, such as the quantity of antibiotics prescribed and rate of antibiotic use, for example prescriptions per patient treated.^17^ Our results show that it is preferrable to report the appropriateness (quality of prescribing) in addition to the quantity of antibiotics prescribed. However, defining appropriateness can be challenging, not least due to guideline differences around the world. For example, prophylactic use of dental antibiotics predominates in some countries (such as in the US), whereas in others therapeutic use to treat infections is most common (for example in the UK).^4^ Consensus on a set of 32 quality indicators measuring appropriateness of antibiotic prescribing across health care settings globally has been published, based on a systematic review followed by a structured consensus process.^22^ Although encompassing a wide range of conditions, none are oral or dental conditions and many (such as those relating to outpatient parenteral antibiotic therapy) are not applicable to dentistry. Further work to operationalise COS-DABS should take account of these internationally agreed metrics on quantity and quality of antibiotic prescribing.

Comparing dental antibiotic use amongst countries is further frustrated by differences in models of oral health care service delivery.^23^ Some dental ABS studies have used routinely collected data from health care systems.^24^ Ideally such systems would be linked between medicine and dentistry to enable collection of all outcome data for the COS, such as adverse outcomes requiring emergency medical care. Further research is required to precisely define the COS-DABS indicators to be used during clinical trials and to explore the feasibility of collecting these data directly from health care systems.

Assessment of stewardship outcomes at an individual patient level, rather than the more common cluster level, has been previously recommended.^18^ The patient-reported outcome included within the COS was derived from the standardised core set of outcomes for adult oral health.^25^ Further work to operationalise this outcome for studies of dental AMS should take into account the SPIRIT-PRO guidance about including patient-reported outcomes in clinical trials.^26^^,^^27^ The authors are also aware that European Union–funded research is currently under way, building on a recent systematic review,^28^ to develop ways to improve the quality of oral health care including by routinely collecting patient feedback.^29^ These patient-reported data might prove useful for researchers using COS-DABS as well as for future work to develop a COS for DABS in other settings, such as for use in routine oral health care and dental services.

An important limitation on this study to achieve international consensus was restricted international travel due to the COVID-19 pandemic. Whilst every effort was made to achieve equal representation of each group,^30^ variable rates of recruitment and loss to follow-up occurred in each group. As described in the results section, last-minute cancellations before the final consensus meeting meant there were fewer patient/parent/carer participants than defined in the protocol. The decision to reschedule the meeting was considered, but there was a risk that even fewer participants would attend a future meeting, so the planned meeting went ahead, with mitigation (as described earlier) to ensure equity of voice for all participant groups.

A pragmatic choice had been made in the protocol about the number of participants to include in each phase of the study, as consensus does not exist in the literature on an appropriate sample size for COS studies.^11^ Whilst reflecting the small size of the community of interest in dental antibiotic stewardship, this is a limitation of the study's method. Even with a larger number or different demographic of participants, however, the authors would not expect the results to change as there was clear separation between the outcomes to be included in COS-DABS and the other candidate outcomes score. The included outcomes were scored as critical by 73% to 100% of the participants, compared to 27% to 58% for the other candidate outcomes. Whilst notable differences exist in the scores between the groups of participants for the patient-reported outcomes, none of the excluded outcomes was scored as “critical for inclusion” by any of the groups.

Due to COVID-19 restrictions, an online approach to recruitment to the study and to the final consensus meeting was selected, which became a strength as it resulted in a more diverse mix of clinician, academic, and patient participants from around the world than would have been possible with face-to-face meetings. Whilst this online approach may have restricted the type of people who would participate, recruitment of research participants via social media, such as Twitter, is increasingly common.^31^ Participants at the final consensus meeting felt inclusion of colleagues from around the world across the 3 stakeholder groups had successfully ensured that a wide range of cultures and perspectives were included.

Whilst the study was conducted entirely in English, the study steering group was keen to ensure that participants from nations where English is not a first language were involved. This introduced a further limitation about whether all participants fully understood what was being asked of them and further research to explore the impact of this is indicated.^32^ A related strength, however, was the inclusion of participants from a diversity of countries, including 8 low- and middle-income countries.

## Conclusions

Antibiotics are an important part of a dentist's armamentarium in keeping patients safe from infections, but they bring with them their own risks. This COS for use in studies about dental ABS presents the ideal set of outcomes to be reported by all studies of dental ABS and will enable maximisation of the benefits which can be derived from future international studies of ABS. Demonstrating reduced antibiotic prescribing without compromising patients safety or quality of life will be a powerful argument for the significant contribution of oral health professionals to international efforts to tackle antimicrobial resistance through judicious antibiotic prescribing.

## Conflict of interest

None disclosed.
